# A New Hematocrit Measurement Method Using a Chemiluminescence Biosensor and Its Application in a Chemiluminescence Immunoassay Platform for Myocardial Markers Detection with Whole Blood Samples

**DOI:** 10.3390/bios13010003

**Published:** 2022-12-21

**Authors:** Huan Zhao, Hao Han, Qifeng Lin, Li Huang, Xiangyi Su, Yile Fang, Yuanying Zhang, Enben Su, Zhu Chen, Song Li, Yan Deng, Nongyue He

**Affiliations:** 1 State Key Laboratory of Bioelectronics, School of Biological Science and Medical Engineering, Southeast University, Nanjing 210096, China; 2 Getein Biotechnology Co., Ltd., Nanjing 210000, China; 3 Department of Molecular Biology, Jiangsu Cancer Hospital, Nanjing 210009, China; 4 Hunan Key Laboratory of Biomedical Nanomaterials and Devices, Hunan University of Technology, Zhuzhou 412007, China

**Keywords:** hematocrit, chemiluminescence, whole blood, correction formula, myocardial markers

## Abstract

The accuracy and precision of analyte concentrations measured in whole blood by chemiluminescence immunoassay (CLIA) have been significantly affected by erythrocytes, which leads to poor application of whole blood CLIA in clinical practice. In this work, a chemiluminescence biosensing optical platform for blood hematocrit (HCT) analysis using MAGICL 6000 (Getein Biotechnology, Nanjing, China) was designed, implemented, and fully characterized. The developed method was successfully applied to determine various HCT levels of human blood from 0% to 65%, with a correlation coefficient of 0.9885 compared with the conventional method (Sysmex XE 5000, Kobe, Japan). A mathematical model was developed to quantitatively evaluate the impact of HCT on the results of two sample types (whole blood vs. plasma). Combining the established HCT method and mathematical model with CLIA on MAGICL 6000, the precision was significantly improved by almost 20%. Comparison studies using whole blood samples and corresponding plasma samples showed that the square of the correlation coefficients of troponin I (cTnI), myoglobin (MYO), creatine kinase MB (CK-MB), and N-terminal pro-hormone brain natriuretic peptide (NT-proBNP) were increased to 0.9992, 0.9997, 0.9996, and 0.9994, respectively, showing a great potential for clinical application.

## 1. Introduction

The ratio of the packed red blood cell volume to the total volume of blood, known as hematocrit (HCT, %), provides critical information about a patient’s health status that helps physicians to diagnose and treat various medical conditions, ailments, and diseases [1,2,3]. The normal HCT ranges of women and men are from 36 to 45% and from 39 to 50%, respectively [4]. In the clinical laboratory, multiple techniques ranging from simple (e.g., centrifugation) [5,6] to sophisticated (e.g., automated analyzers) [7,8] are used to determine HCT values, and the manual spun HCT is considered as the reference method [7,9]; however, the chemiluminescence biosensor method has not been reported. The established HCT detection method for human blood samples reported in this paper requires only 0.1 μL of sample loading without any sample pretreatment, which is much easier than the traditional method. The pre-trigger solution not only provides the environment to be excited, but also acts as a hemolysis agent to release heme as a luminescent substrate in the blood cells. With the injection of the trigger solution, the substrate reacts with hydrogen peroxide to generate light through a chemical reaction. Therefore, the chemiluminescence method has the advantages of ultra-high sensitivity and extremely short detection time, which can be completed within 3 s.

Chemiluminescence immunoassay (CLIA) combined a chemiluminescent system with immunoreaction is one of the most widely used biomedical diagnostic methods due to its sensitivity and specificity [10,11,12,13]. The on-site detection of myocardial infarction from human body liquids such as whole blood is highly desirable, particularly in resource-limited settings, because of the increasing demand for early diagnostics and rapid testing results [14,15]. Since the adoption of reporting whole blood concentrations of CLIA, several studies have revealed that HCT levels influence the accuracy of an immunoassay for analyte determination [16,17,18]. These studies were conducted because the HCT values of hospitalized patients often deviate from the mid-point of the reference interval: neonatal patients often have higher or lower hematocrit levels and the hematocrit levels of adult critical care and chronic renal disease patients are often lower. Although the manufacturers integrate the HCT measuring module in automated CLIA analyzers for concentration detection in whole blood samples, and the HCT values are used to correct the plasma equivalent concentrations [10,13,19,20], little attention has been paid to describing the nature of the HCT influence or the expected mathematical relationship or model between HCT and the reported sample concentration. Meanwhile, the combination of HCT analysis and CLIA in whole blood immunoassay not only delays the detection time, but also increases the manufacturing cost, due to the common HCT measurement methods relying on prolonged manual labor or the use of a power source. To perform the HCT analysis more cheaply, they require the purchase of improved instruments in addition to the consumables needed to perform a separation or analysis The proposed method can not only be used to report the patient’s HCT results, but can also be integrated into a CLIA analyzer for the plasma equivalent correction of whole blood, which can significantly improve the accuracy of the immunoassay for whole blood samples without increasing the cost.

Troponin I (cTnI), myoglobin (MYO), and creatine kinase MB (CK-MB) assays provide enhanced detection of myocardial injury and potentially improve the efficiency of the diagnosis and management of acute myocardial infarction (AMI) patients [14]. Studies indicate that N-terminal pro-hormone brain natriuretic peptide (NT-proBNP) can be used in diagnostic and prognostic applications [21,22,23]. The concentration of NT-proBNP in serum or plasma correlates with the prognosis of left ventricular dysfunction. It was found that congestive heart failure patients with NT-proBNP values above median had a one-year mortality rate of 53% compared to 11% in patients below median [24]. Several detection methods, with most research focusing on the improved electrochemiluminescence method, have been fabricated for the determination of AMI biomarkers in plasma or serum [25,26,27,28]. However, whole blood seems to be clinically preferred, because it offers advantages in an emergency room or other near-patient settings where the time to obtain the results of the aforementioned myocardial markers and user convenience is critical. Moreover, some of these methods achieved high sensitivity and good selectivity with multiple steps by a professional person to operate complex instruments, which significantly restricted their applicability for point-of-care testing (POCT).

POCT techniques are currently investigated and applied in many fields [29,30,31,32]. Different POCT devices have been reported [33,34,35,36,37,38]: some of these strategies improved analytical performance through complex preparation processes [39,40,41,42,43], some through the application of new nanomaterials [44,45,46,47,48], while others were to simplify equipment and report detection results quickly without guaranteeing high performance [49,50,51,52,53]. How to improve the analysis performance of rapid detection while simplifying the device and shortening the detection time has always been a challenge. Based on our previous studies [11,12,13], the objectives of this study were to establish a new method for the rapid quantitative measurement of HCT by a chemiluminescence biosensor using a CLIA-based platform, and to develop a mathematical model based on the mathematical relationship between HCT and reported whole blood sample concentration. Finally, the application of this HCT measurement and mathematical model for the detection of myocardial markers with whole blood samples on Getein^®^ MAGICL 6000 was investigated.

## 2. Materials and Methods

### 2.1. Getein^®^ CLIA for Myocardial Markers Detections

Getein^®^ MAGICL 6000 is a fully automated piece of diagnostic equipment that uses CLIA technology to determine the presence of antigens, antibodies, and analytes in samples. The reactants necessary to perform CLIA assays include paramagnetic microparticles coated with a capture molecule specific for the analyte being measured and acridinium-labeled conjugate used to form an immunocomplex sandwich. The trigger (alkaline) and pre-trigger (peroxide) solution create an oxidative reaction when combined with acridinium conjugate. The specimens, including human whole blood, plasma, and serum, were tested and found acceptable for a variety of myocardial marker detections such as cTnI, MYO, CK-MB, and NT-proBNP on the analytical instrument. With whole blood samples, the analyzer’s reader subsystem measured HCT at each sampling. HCT correction was calculated by a software algorithm, and finally, the results were reported accurately in whole blood. The biological principles of the procedure are as follows: a certain volume of whole blood samples was lysed with the denaturing agents containing 20 mmol/L CTAB and 1 mol/L ammonium chloride (no pretreatment was required for serum or plasma), and then incubated at 37 °C with microparticles coated capture antibodies and acridinium-labeled detection antibodies. After 7 min incubation, the resulting immunocomplexes were separated, washed to remove unbound substances, and then transferred to an optical detection vessel for reading with the MAGICL 6000 counting detection system. The results are determined via a calibration curve, which is instrument-specifically generated by two-point calibration and a master curve provided via the reagent barcode. The commercial cTnI, MYO, CK-MB, NT-proBNP CLIA kit was provided by Getein Biotechnology. The chemicals were purchased from Sigma-Aldrich (St. Louis, MO, USA) at analytical reagent grade, and deionized, doubly distilled water was used throughout.

### 2.2. Sample Preparation

The blood samples of 58 healthy people used for HCT analysis by a chemiluminescence biosensor and 86 AMI patients used for myocardial markers immunoassay were obtained from Xuzhou Central Hospital, and approved by Xuzhou Central Hospital Research Ethics Committee. To evaluate the potential impact of the red blood cells on CLIA, plasma with EDTA-K2 as an anti-coagulant and the matched whole blood were used. The EDTA-K2 plasma samples were used after centrifugation and whole blood samples were used within 2 h.

### 2.3. HCT Analysis by Chemiluminescence Biosensor

Multiple methods exist for the determination of HCT in the clinical laboratory. However, the chemiluminescence biosensor method has not been reported. A total of 100 mL collected whole blood was transferred to eight 10 mL polypropylene transparent tubes and manually reconstituted to eight different HCT levels of 0, 25, 30, 35, 40, 45, 50, 55, and 60% by either adding plasma to the centrifuged whole blood samples or removing it. A desired range of HCTs was prepared to generate the standard calibration curve. The chemiluminescence method on the MAGICL 6000 analyzer was as follows, after adding 2 μL of the prepared whole blood sample into the reaction cup, the pre-trigger solution containing 0.5% W/V hydrogen peroxide and 0.2 mol/L nitric acid, and the trigger solution containing 0.35 mol/L sodium hydroxide and 0.2% W/V CTAC were added to excite heme in blood cells as the substrate; the resulting chemiluminescent reaction was measured as relative light units (RLUs). The excitation conditions were consistent with the process of acridinium conjugate excitation in immunoassay. A direct relationship exists between the amount of red blood cells in the sample and the RLUs detected by the photomultiplier tube optical system.

To assess the effect of potentially interfering substances on hematocrit measurement, a phosphate buffer solution as a negative sample and four potentially interfering substances at high concentrations (human serum albumin, 500.0 mg/dL; triglycerides, 1000.0 mg/dL; bilirubin, 50.0 mg/dL; total cholesterol, 2000.0 mg/dL) were added in fixed ratios (10/0, 9/1, 8/2, 7/3, 6/4, 5/5, 4/6, 3/7, 2/8, 1/9, 0/10) to generate the samples containing gradient concentrations of interfering substances. The whole testing was performed in duplicate, and the interference was evaluated by calculating the deviation of the RLUs between potential interferent-spiked samples and unspoked (no potential interferent) samples. No interference was defined as ≤10% difference for the average of all RLUs tested.

In order to evaluate the proposed method as a platform for the rapid determination of HCT in patient samples, 58 EDTA-treated whole blood samples were collected by convenience sampling depending on research staff and sample availability. Following sample mixing, HCT was measured both on the MAGICL 6000 analyzer by a chemiluminescence biosensor, as mentioned above, and on the Sysmex XE 5000 by impedance according to the manufacturer’s instructions, respectively, within a maximum of 2 h.

### 2.4. HCT Correction Formula for Whole Blood

The relationship between HCT and analyte concentration in the whole blood and plasma was described in a previous literature report and is shown in Equation (1) [10,13,19]. This equation describes that the concentration of analyte in the whole blood, as detected by immunoassay, can be converted into the analyte in plasma. However, it is found that this formula has some limitations in practical applications, especially at lower HCT (<30%) or higher HCT (>60%), indicating that HCT not only affects plasma volume, but also alters the immunobinding rate.

cTnI _plasma_ = cTnI/(1 − (HCT _whole blood_/100) (1)

To investigate the influence of the HCT, reconstituted blood samples at different HCT levels of 0, 25, 30, 35, 40, 45, 50, 55, and 60% consistent with the above preparation methods were gently mixed with cTnI standard using a tube shaker to disperse the analyte evenly within the blood samples. cTnI was added in five concentration gradients ranging from 0.1 to 50 ng/mL. The 45 samples prepared were tested on a MAGICL 6000 analyzer for deviation analysis.

### 2.5. Application of HCT Correction for Immunoassay

#### 2.5.1. Repeatability Study

The repeatability study was assessed with the patient specimens following CLSI guideline EP5-A3 on the MAGICL 6000 chemiluminescence analyzer. Levels 1–3 of three whole blood samples with plasma equivalent levels were assayed 10 times to assess the repeatability by the Getein^®^ CLIA cTnI, MYO, CK-MB, NT-proBNP reagents. The mean, SD, and CV% were calculated.

#### 2.5.2. Accuracy Study

Plasma and the whole blood sample types were collected from each patient to evaluate the potential impact of the blood cells and HCT correction on the measured concentration of CLIA. Eighty-six paired samples with detectable myocardial marker concentrations obtained from AMI patients were analyzed using the MAGICL 6000 assays. cTnI, MYO, CK-MB, and NT-proBNP were measured in two sample types within 2 h following collection. The results in the whole blood samples were obtained after software correction for individual HCT values according to the correction formula. The regression equation and correlation coefficient were performed to evaluate the correlation between the two sample types, and the mean relative error was estimated by the Bland–Altman method to determine the accuracy of the whole blood samples.

### 2.6. Statistical Analysis

With Microsoft Excel 2007, the results were tabulated. Method comparisons were performed using MedCalc software (v.19.5.6) via Passing Bablok regression and the Bland–Altman plot. Statistical analysis on a completely randomized design was conducted using the one-way analysis of variance (ANOVA) procedure with SPSS software (v.23.0). Numerical data were expressed as mean ± standard deviation (SD). Measured values from plasma samples were compared with those obtained from the whole blood samples using a paired-sample t-test. Statistical significance was considered at the level of *p* < 0.05.

## 3. Results and Discussion

### 3.1. HCT Measurement

**Figure 1:**
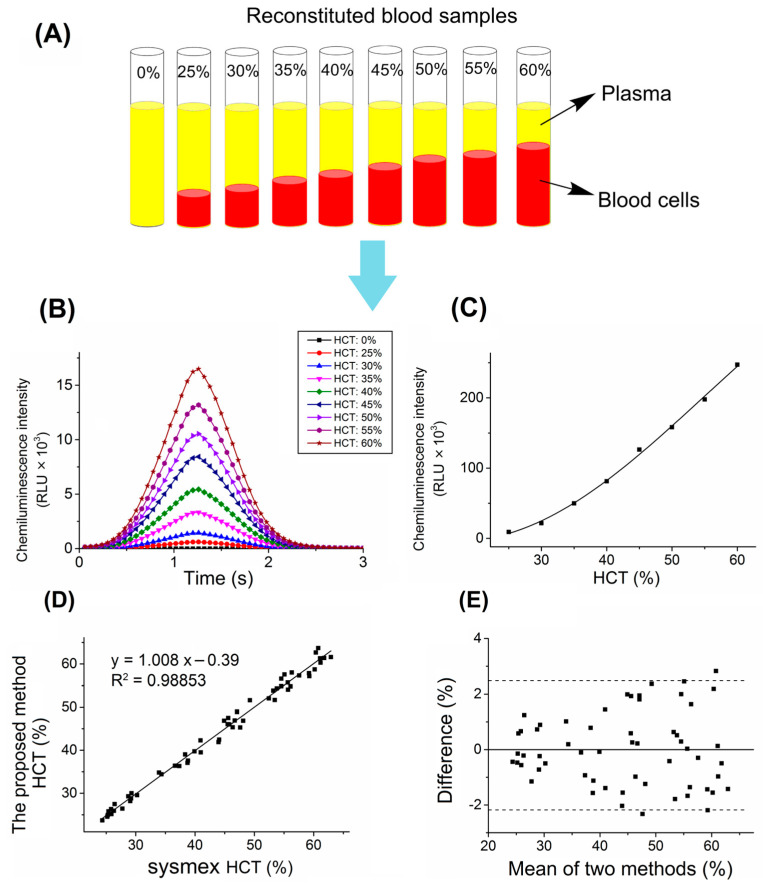
(**A**) Procedure to reconstitute whole blood to target HCT levels ranging from 0 to 60%. (**B**) Chemiluminescence curves of hemoglobin in the reconstituted blood samples at different HCT levels were generated by MAGICL 6000. (**C**) Standard curves were obtained by the chemiluminescence biosensor method for HCT measurement. Comparison study between the established method and the clinical method (Sysmex XE 5000) for HCT measurement. (**D**) Correlation analysis; (**E**) Bland-Altman analysis.

To characterize the correlation between the blood HCT and chemiluminescent intensity triggered by hydrogen peroxide and sodium hydroxide solutions, the reconstituted blood samples at different HCT levels of 0, 25, 30, 35, 40, 45, 50, 55, and 60% (Figure 1A) were prepared to observe the chemiluminescence curve with a MAGICL 6000 analyzer. As shown in Figure 1B, at the optimal conditions, the maximum number of photons occurred at 1.26 s after being triggered, and the total number of photons is linearly proportional to the amount of red blood cells. Figure 1C shows that a representative standard curve (RLUs values against HCT levels of 25~60%) was successfully obtained with a correlation coefficient of 0.990. This chemiluminescence sensor method established successfully is shown not only to provide accurate quantitative HCT measurement, but also to perform so substantially faster (3 s) with less sample volume (2 μL) than the conventional measurement method. The principle has been described in previous papers; briefly, hemoglobin in red blood cells containing four iron heme groups acts as a substrate for chemiluminescence reaction in the presence of hydrogen peroxide to produce photons [12,54,55].

To determine the specificity of the HCT assay, endogenous interfering substances were investigated. No significant interferences were observed with human serum albumin concentrations at 500.0 mg/dL; triglycerides at 1000.0 mg/dL; bilirubin at 50.0 mg/dL; and total cholesterol at 2000.0 mg/dL. To evaluate the accuracy of the chemiluminescence biosensor method, the HCT analysis of EDTA-whole blood samples was compared with HCT values determined on a Sysmex XE 5000 analyzer with electronic resistance detection in the clinical lab. A method comparison for HCT testing was performed by Passing Bablok regression and the Bland–Altman analysis method, and the results are shown in Figure 1D,E. The regression equation of Sysmex value (x) and the proposed method value (y) had a slope of 1.008, intercept of −0.39, and correlation coefficient of 0.98853, and the Bland–Altman analysis showed that the 95% confidence interval of the mean absolute bias was between −2.55% and 2.63%, demonstrating an excellent correlation between the two methods.

### 3.2. Effect of HCT on CLIA

**Figure 2:**
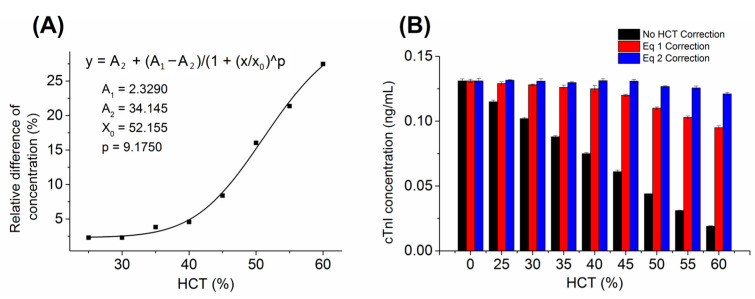
(**A**) Standard curve of relative difference of the concentration of cTnI vs. HCT value. (**B**) HCT effect for cTnI CLIA detection.

The HCT value is discussed as a factor influencing the analyte concentrations of the immunoassay. Therefore, blood samples with HCT values ranging from 0 to 60% were evaluated for their effect on cTnI quantification. In detail, one blood sample at the HCT of 43% containing an average cTnI concentration of 0.125 ng/mL was adjusted to eight different HCT values (25, 30, 35, 40, 45, 50, 55, and 60%) and analyzed. The results of the HCT effect for cTnI CLIA detection are shown in Figure 2. The determined cTnI concentrations showed a significant difference (*p* > 0.05) compared with the plasma sample (HCT = 0%), indicating that the HCT value had a strong influence on cTnI analysis. After correction by Equation (1), the accuracy was effectively improved for HCT between 25% and 40%. However, the correction formula was not applicable for higher HCT values, indicating that the difference in cTnI concentration in high HCT samples was not only due to the reduction of available plasma volume, but also due to the reduction of excessive cell debris in the reaction system, which slowed the rate of immune binding. Figure 2A was created to describe the influence of HCT on the cTnI concentration of the whole blood sample and showed that the deviation tended to be larger when the HCT is higher, and a four-parameter logistic curve fit method was utilized to generate a calibration curve with a correlation coefficient of 0.994. The fitting equation was:

(2) $$y=34.145-\frac{31.816}{{\left(1+\frac{x}{52.155}\right)}^{9.175}}$$

x: HCT values (%); y: relative difference of cTnI concentration (%).

As shown in Figure 2B, the mean recovery was 51.0% with values ranging from 14.5% to 87.8% without HCT correction; the mean recovery was 89.3% with values ranging from 72.5% to 92.5% with Equation (1) correction; and the mean recovery was 98.0% with values ranging from 92.3% to 100.4% with Equation (2) correction. The recovery in the whole blood sample was significantly improved after the secondary compensation using Equations (1) and (2), indicating that the interference of red blood cells in the process of immune binding could be compensated by the proposed mathematical model. It was concluded that non-linear regression is an informative strategy to quantify the influence of HCT on whole blood CLIA analytical devices.

### 3.3. Repeatability Testing

The analytical performances of cardiac markers including cTnI, MYO, CK-MB, and NT-proBNP were evaluated on a MAGICL 6000 chemiluminescence analyzer. For precision analysis, three whole blood samples were tested 10 times with or without HCT correction on the days of sample collection. Their average concentrations were 0.114, 0.522, and 5.471 ng/mL for cTnI, 120.2, 588.1, and 2023.1 ng/mL for MYO, 10.1, 52.8, and 155.0 ng/mL for CK-MB, and 128.1, 5152.6, and 21006.8 pg/mL for NT-proBNP, respectively. As shown in Table 1, the observed assay repeatability (CV) at 95% confidence limits ranged from 1.85% to 3.13% for various cTnI concentrations, 1.80% to 3.01% for various MYO concentrations, 1.99% to 3.32% for various CK-MB concentrations, and 1.88% to 3.07% for various NT-proBNP concentrations. Correspondingly, the repeatability under control conditions without HCT correction ranged from 2.21% to 3.82% for cTnI, 2.13% to 3.87% for MYO, 2.48% to 4.53% for CK-MB, and 1.85% to 3.99% for NT-proBNP. Compared with the control group without considering the influence of red blood cells, the precision was significantly improved by almost 20%. The repeatability of whole blood samples in CLIA has been one of the major challenges—because of the heterogeneity of samples caused by erythrocyte sedimentation, the effective plasma volume of each sampling process is inconsistent. HCT values tested using the proposed method enhanced the repeatability due to eliminating the influence of erythrocyte inhomogeneity during the mixing and sampling of the whole blood samples.

### 3.4. Accuracy Testing

**Figure 3:**
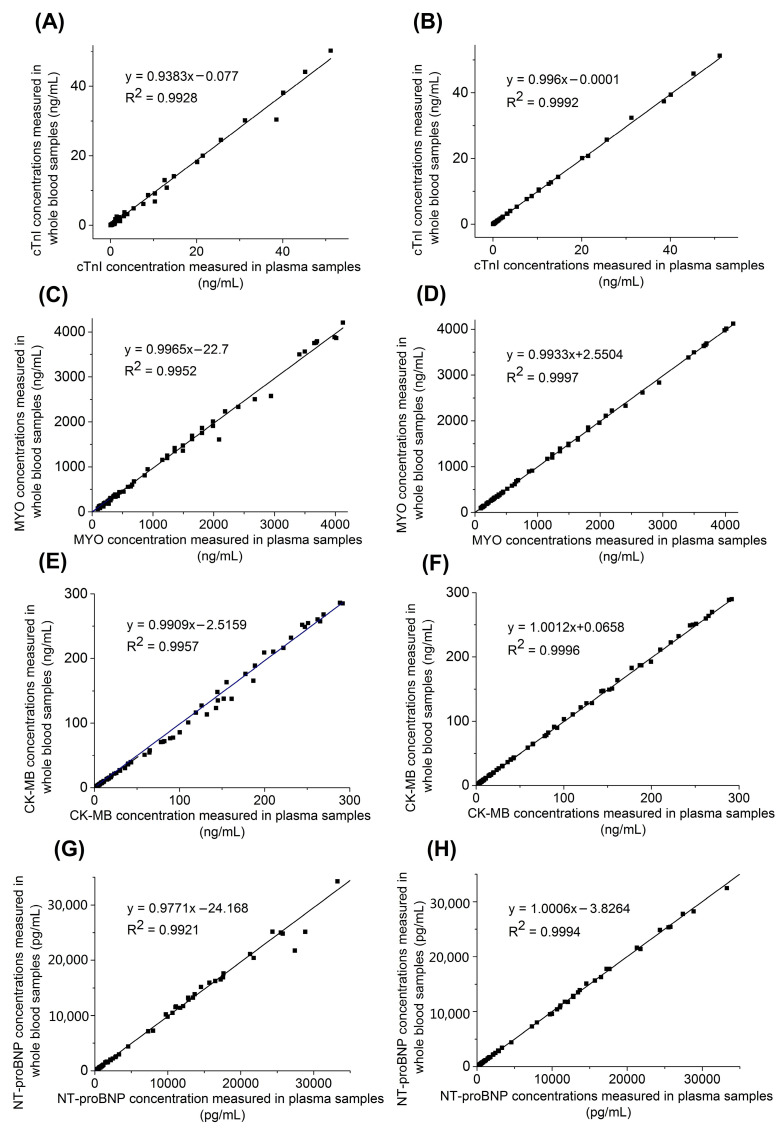
Comparison studies using whole blood samples and corresponding plasma samples for cTnI detection without HCT correction (**A**) and with HCT correction (**B**), for MYO detection without HCT correction (**C**) and with HCT correction (**D**), for CK-MB detection without HCT correction (**E**) and with HCT correction (**F**), for NT-proBNP detection without HCT correction (**G**) and with HCT correction (**H**).

The concentrations of myocardial markers in the whole blood samples and corresponding plasma samples were compared to assess the potential effect of red blood cells and HCT correction on accuracy. As shown in Figure 3A,C,E,G, myocardial marker concentrations measured with two sample types on the MAGICL 6000 yielded the square of the correlation coefficient of 0.9928 for cTnI, 0.9952 for MYO, 0.9957 for CK-MB, and 0.9921 for NT-proBNP. The Bland–Altman analysis showed that the mean relative difference was −14.2% for cTnI, −7.8% for MYO, −8.66% for CK-MB, and −7.69% for NT-proBNP. Using the mathematical model Equation (2) for HCT correction, Figure 3B,D,F,H showed that the square of the correlation coefficients of cTnI, MYO, CK-MB, and NT-proBNP were increased to 0.9992, 0.9997, 0.9996, and 0.9994, respectively. The mean relative differences of cTnI, MYO, CK-MB, and NT-proBNP were −0.4%, −0.79%, 0.17%, and 0.35%, respectively, from the Bland–Altman plot results, indicating that the concentrations measured in the whole blood samples with HCT correction had better consistency with the matched plasma samples. The enhanced CLIA method on the MAGICL 6000 for whole blood, which simultaneously measures HCT and performs automated correction for the HCT effect, provides myocardial marker results with improved accuracy. Its measurement of HCT from the same blood sample will eliminate the need for the additional collection of blood or measurements using another method, which has great application prospects in the clinical application of CLIA whole blood analysis.

## 4. Conclusions

In this work, the rapid quantification of blood HCT was successfully completed by a chemiluminescence biosensor using a CLIA-based platform. To demonstrate the accuracy of this technique, 58 clinical samples were analyzed in our laboratory with the results correlating very well with the clinical analyses (Sysmex XE 5000). The study of HCT influence on cTnI immunoassay indicated that increased HCT impaired immune binding when analyzed in whole blood. With Equation (2) established by a four-parameter logistic curve fit method, the recovery in the whole blood sample was significantly improved. Combining this HCT measurement and correction with CLIA, which shares the same excitation conditions and photon acquisition device on the MAGICL 6000, we can directly detect the myocardial marker concentrations in the whole blood samples without the need for other HCT measurement instruments. According to the performance analysis, the established method has excellent reproducibility and accuracy due to the advantages of HCT correction, which avoids the influence of erythrocyte inhomogeneity on repeated sampling and excessive cell debris on immune binding.

## Figures and Tables

**Table 1:** cTnI/MYO/CK-MB/NT-proBNP detection repeatability of whole blood samples at 95% confidence limits.

Samples		cTnI	MYO	CK-MB	NT-proBNP
Level 1	Control	2.72–3.82%	2.58–3.87%	2.95–4.53%	2.20–3.99%
	HCT Correction	1.87–3.13%	1.81–3.01%	1.99–3.29%	1.90–3.07%
Level 2	Control	2.44–3.46%	2.35–3.68%	2.80–4.19%	1.85–3.61%
	HCT Correction	1.91–2.99%	1.83–2.98%	2.06–3.29%	1.89–2.98%
Level 3	Control	2.21–3.13%	2.13–3.32%	2.48–3.91%	2.50–3.14%
	HCT Correction	1.85–2.98%	1.80–2.98%	2.05–3.32%	1.88–2.97%

## Data Availability

All data are contained within the article.
